# A haplotype-resolved genome provides insight into allele-specific expression in wild walnut (*Juglans regia* L.)

**DOI:** 10.1038/s41597-024-03096-4

**Published:** 2024-03-08

**Authors:** Liqun Han, Xiang Luo, Yu Zhao, Ning Li, Yuhui Xu, Kai Ma

**Affiliations:** 1 Institute of Horticulture Crops, Xinjiang Academy of Agricultural Sciences, the State Key Laboratory of Genetic Improvement and Germplasm Innovation of Crop Resistance in Arid Desert Regions, Key Laboratory of Genome Research and Genetic Improvement of Xinjiang Characteristic Fruits and Vegetables, Urumqi, China; 2 College of Agriculture, Henan University, Zhengzhou, China

**Keywords:** Plant breeding, Plant stress responses

## Abstract

Wild germplasm resources are crucial for gene mining and molecular breeding because of their special trait performance. Haplotype-resolved genome is an ideal solution for fully understanding the biology of subgenomes in highly heterozygous species. Here, we surveyed the genome of a wild walnut tree from Gongliu County, Xinjiang, China, and generated a haplotype-resolved reference genome of 562.99 Mb (contig N50 = 34.10 Mb) for one haplotype (hap1) and 561.07 Mb (contig N50 = 33.91 Mb) for another haplotype (hap2) using PacBio high-fidelity (HiFi) reads and Hi-C technology. Approximately 527.20 Mb (93.64%) of hap1 and 526.40 Mb (93.82%) of hap2 were assigned to 16 pseudochromosomes. A total of 41039 and 39744 protein-coding gene models were predicted for hap1 and hap2, respectively. Moreover, 123 structural variations (SVs) were identified between the two haplotype genomes. Allele-specific expression genes (ASEGs) that respond to cold stress were ultimately identified. These datasets can be used to study subgenome evolution, for functional elite gene mining and to discover the transcriptional basis of specific traits related to environmental adaptation in wild walnut.

## Background & Summary

Common walnut (*Juglans regia* L.) (2n = 32), which belongs to the *Juglans* genus of Juglandaceae, is an important economic tree species and the source of the fourth most consumed nut^1^. The fruits of common walnut are mainly used for the production of oil and health care products due to their lipid profile and abundance of antioxidants, such as phenolic compounds, vitamins and micronutrients^2–4^, which have potential beneficial roles in health maintenance and disease prevention^5,6^. These properties have promoted walnut cultivation and production in many countries, such as the USA, Iran, Turkey and China^7^.

Walnut likely originated and was domesticated in Central Asia^8,9^ and has been found in China for more than 7000 years^10^. Currently, China is regarded as a secondary centre of origin, harbouring wide genetic diversity^11,12^. Wild walnut is widely distributed in a nature reserve in Gongliu County, Xinjiang, China^13–15^. Wild relatives often harbour elite alleles conferring tolerance to extreme biotic or abiotic stress in specific environments^16–18^, which can provide resistance resources for genetic improvement. Xinjiang wild walnut shows relatively high stress resistance^19,20^ and good nutritional components^21^. Specifically, the elite cold resistance of wild walnut can guarantee its survival and propagation in long-term extreme cold, whether in winter or late spring, in Gongliu County. Therefore, wild walnut in this area is an ideal material for cold-improvement molecular breeding and the identification of cold stress regulatory mechanisms. However, walnut is a nonmodel tree species, and most studies have focused on the quality of cold-stressed walnut kernels^22,23^ or phenotyping the cold resistance of walnut varieties^24^, with few focusing on the molecular mechanism of cold tolerance^25^.

The draft genome of walnut was released in 2016^26^ and improved in 2018^27^. Since then, reference genome assemblies have been published successively with higher genome continuity or at the chromosome scale by means of long-read sequencing and Hi-C anchoring^28,29^. In addition, the genome sequences of six species of Juglandaceae (*J. sigillata*, *J. cathayensis*, *J. mandshurica*, *J. hindsii*, *J. microcarpa* and *J. nigra*^30^) are also available (http://xhhuanglab.cn/data/juglans.html). However, the walnut genome has high heterozygosity between diploid chromosomes^27–29^, and the current genomes have mosaic assemblies of haplotypes. Recently, haplotype-defined genome assembly was realized in highly heterozygous organisms such as *Manihot esculenta*^31^, *Camellia sinensis*^32^, *Artemisia annua*^33^ and *Malus* spp.^34^, which provided novel insight into allelic variation calling and functional differentiation of divergent alleles in heterozygous species.

Diploid organisms have two allelic copies in their genome, but in a given individual, the alleles are not necessarily both active or active at the same level. This unequal expression of gene copies caused by cis-acting genetic variants or CHG methylation is called allele-specific expression (ASE)^35,36^. ASE was first reported in yeast in 2002^37^ and is involved in many plant biological processes, such as the response to mild water deficit in tomato^38^ and heterosis via partial to full dominance or overdominance effects on the traits being regulated and chromatin accessibility alterations in rice^39,40^. In recent years, genome-wide ASE features and ASE-related genes have been well characterized, benefitting from haplotype-resolved genome assembly in this third-generation sequencing era^41,42^. In the woody perennial plant apple, approximately 19% of the expressed genes were allele specific, and many ASE genes, such as genes encoding ACC oxidase and RIN-like MADS-box transcription factors, were associated with fruit quality^43^. Furthermore, Tian *et al*.^34^ reported that transposable elements (TEs) can regulate ASE to determine flower colour in apple.

This study reports a high-quality haplotype-resolved reference genome of wild walnut. First, Illumina sequencing reads were used to estimate the genome size, heterozygosity and proportion of repetitive sequences. PacBio high-fidelity (HiFi) long reads were adopted to primarily assemble highly continuous contigs, followed by HiFi read phasing to reassemble the resulting haplotypes. Then, two haplotypes (hap1 and hap2) at the chromosome scale were identified by Hi-C technology-assisted assignment. The final assembly lengths were 562.99 Mb and 561.07 Mb for hap1 and hap2, respectively, with contig N50 values of 33.91 Mb and 22.40 Mb, respectively. Approximately 93.64% of the contigs (527.2 Mb) were anchored onto 16 pseudochromosomes for hap1, and 93.82% (526.4 Mb) were anchored onto 16 pseudochromosomes for hap2. The LTR assembly index (LAI v2.9.4)^44^, BUSCO v5 completeness^45^, genome collinearity and phasing switch errors^32^ were used to evaluate the completeness, continuity and phasing accuracy of the haplotype genomes. We predicted 41039 gene models for hap1 and 39744 gene models for hap2, and 34617 and 34562 genes were functionally annotated by four conventional databases, respectively. A total of 358869 and 358104 simple sequence repeats (SSRs) were also identified for hap1 and hap2, respectively. The genome variation between hap1 and hap2, including different types of structural variations (SVs), is highlighted. Finally, 358, 457, 465, 494 and 386 ASEGs were identified at 0 h, 3 h, 6 h, 12 h and 24 h after cold treatment, respectively. This high-quality haplotype-resolved genome assembly will shed light on subgenome evolution, molecular genetics and ASE regulatory mechanisms underlying adaptation to the extreme cold environments of wild walnut.

## Methods

### Plant material preparation, Illumina short-read library construction and sequencing

**Fig. 1:**
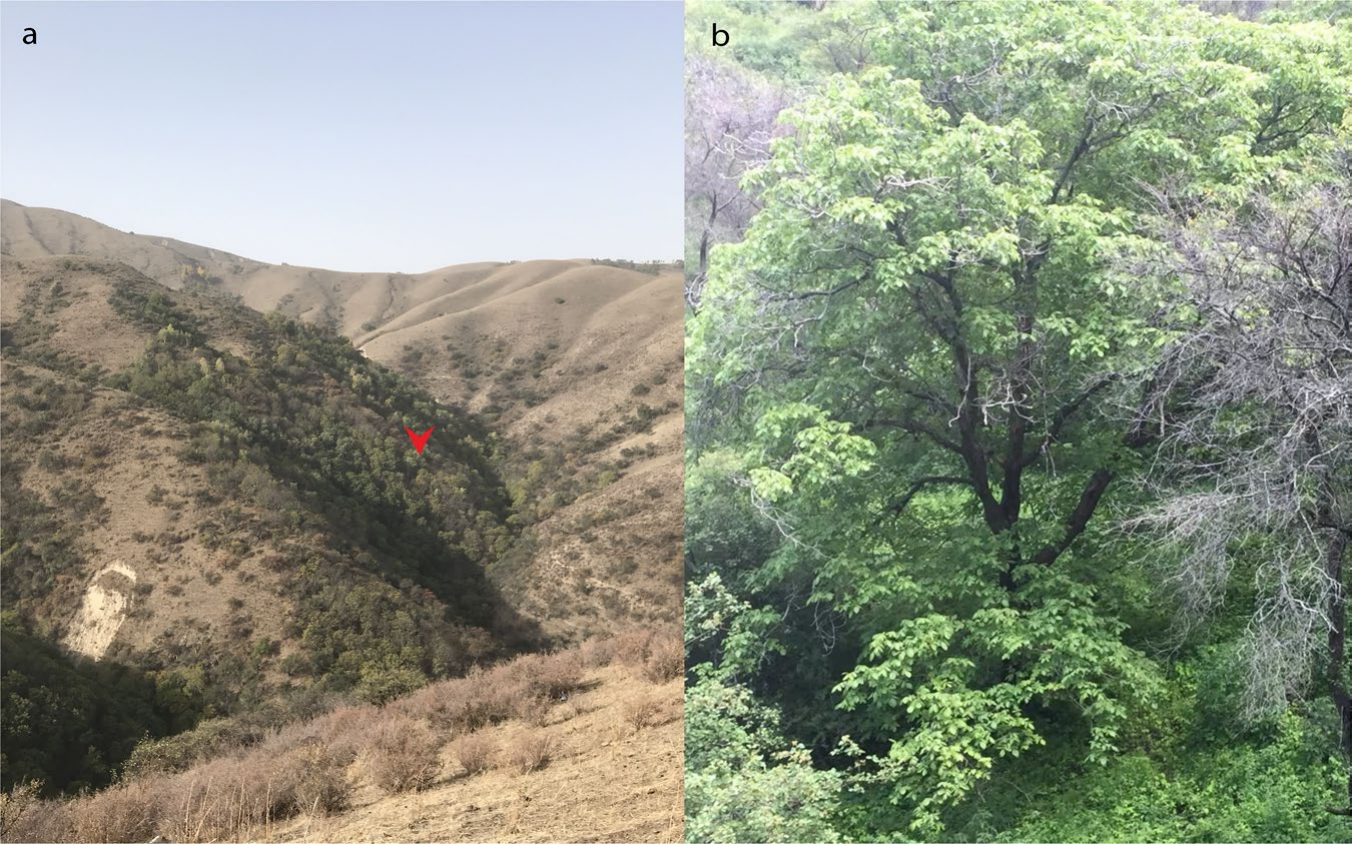
The location and appearance of the wild walnut plants used for this genome assembly. (**a**) The precise location of the sampled wild walnut (red arrowhead). (**b**) The appearance of the sampled wild walnut.

Approximately 20 g of young leaves from a wild walnut tree was collected from the nature reserve of Gongliu County, Xinjiang, China (E82°16′28″, N43°20′52″). The altitude of the wild walnut habitat ranges from 1241 m to 1670 m, and the annual average temperature is 7.6 °C, with an average of 19.7 °C in July and −9.9 °C in January^46,47^. Under cold stress, wild walnut plants exhibit better performance than seedling walnut^48^. The location and appearance are shown in Fig. 1. Then, the leaves were cut into pieces and rapidly placed in liquid nitrogen. The genomic DNA of the fresh leaves was isolated using a Super Plant Genomic DNA DP360 Kit (Tiangen Biotech, Beijing, China), followed by DNA concentration measurement using a NanoDrop spectrophotometer (ND2000, Thermo Fisher Scientific, USA) and quality monitoring by electrophoresis on 0.80% agarose gels. High-quality DNA was subjected to ultrasonic fragmentation (350 bp) using a Covaris S2 instrument (Covaris, Woburn, MA, USA). A 350 bp paired-end resequencing library was prepared using a TruSeq DNA Sample Prep Kit (Illumina, San Diego, CA, USA) according to the manufacturer’s standard protocols. An Agilent 2100 bioanalyzer (Agilent Technologies, Palo Alto, CA) and q-PCR were used to measure the fragment size and quality of the library. To aid in gene model prediction, total RNA was extracted from mixed samples containing leaf, root, shoot and flower tissues (~1 g) from the same tree using the RNeasy Plant Mini Kit (Qiagen, Germany) according to the manufacturer’s instructions and treated with an RNase-free DNase I digestion kit (Aidlab, China). RNA degradation was monitored using a 1% agarose gel, and the purity of the isolated RNA was measured using a NanoDrop 2000 spectrophotometer (Thermo Scientific, Wilmington, DE, USA). The integrity of the RNA was assessed on an Agilent 2100 Bioanalyzer (Agilent, Palo Alto, CA, USA), and the RNA library was constructed with the TruSeq RNA Library Prep Kit v.2 (Illumina, San Diego, CA, USA). Both the DNA and RNA libraries were sequenced on an Illumina NovaSeq. 6000 sequencer (Illumina, San Diego, CA, USA) according to the manufacturer’s recommendations. The raw reads were filtered to generate high-quality clean reads by (i) removing adaptor sequences, (ii) filtering reads with >10% unidentified nucleotides, and (iii) removing reads with >50% bases with a low Phred quality score (≤10). As a result, 31.47 Gb (~56×) and 10 Gb (RNA-seq data) of high-quality clean reads were generated for the genome survey and gene model prediction, respectively.

### PacBio HiFi long-read library generation and sequencing

High-integrity genomic DNA extraction was performed according to Mayjonade’s methods^49^. The HiFi library was used according to PacBio’s standard protocols. Briefly, 10 μg of high-quality gDNA was randomly disrupted by a Covaris g-TUBE device (Covaris, Woburn, MA) and transferred for DNA damage repair and end repair. Then, dumbbell-shaped adaptors were ligated. Thereafter, the products were subjected to exonuclease digestion followed by size selection to screen segments between 10 kb and 30 kb in size using BluePippin (Sege Science, USA). The final library was sequenced on a PacBio Sequel II platform (circular consensus sequencing (CCS) model) using two flow cells for 35 h (Adsen Biotechnology Company, Urumqi, China). Finally, we obtained approximately 57.86 Gb of HiFi reads (~ 103 × depth) with a CCS contig N50 and greatest length of 15.91 kb and 50.33 kb, respectively.

### Hi-C library preparation and sequencing

As living samples can provide integrated and real interaction states *in vivo*, seeds from wild walnuts (for DNA extraction) were collected and germinated indoors. Seedlings at the four-leaf stage were used for Hi-C library preparation according to Lieberman–Aiden’s method^50^. In brief, fresh leaves were immediately crossed with formaldehyde, and the cross-linked DNA was then digested with Hind III (NEB). Subsequently, the sticky ends of these enzyme-digested fragments were end-repaired by a biotin-modified base. Circular DNA molecules were continuously generated using blunt-end proximity-ligated fragments and fragmented into 300–700 bp fragments. Finally, the fragments were enriched by biotin beads, and after library quality control by a Qbit 2.0 (Life Technologies, Carlsbad, CA, US) and an Agilent 2100 bioanalyzer (Agilent Technologies, Palo Alto, CA, USA), the library was subjected to paired-end 150 bp (PE150) sequencing in one lane (Illumina, San Diego, CA, USA). This Hi-C library generated approximately 64.61 Gb (~115 × depth) of clean data.

### Genome survey analysis

A total of approximately 31.47 Gb of clean reads were generated for the genome survey using GenomeScope v1^51^ with the parameters “k = 15, 17, 19, 21, 23; length = 100; and max coverage = 10000”. We obtained an estimated genome size of 470.69 Mb-513.17 Mb with a heterozygosity of 0.29%–0.41% for this wild walnut (Table 1).

**Table 1:** Genome size estimation by k-mer analysis.

k-mer size	Genome size (Mb)	Heterozygosity (%)	Repeat ratio (%)
k = 15	513.17	0.41	32.98
k = 17	509.43	0.30	35.29
k = 19	470.69	0.32	34.12
k = 21	513.17	0.30	33.00
k = 23	484.53	0.29	33.01

### *De novo* assembly of the haplotype-resolved chromosome-scale genome

**Fig. 2:**
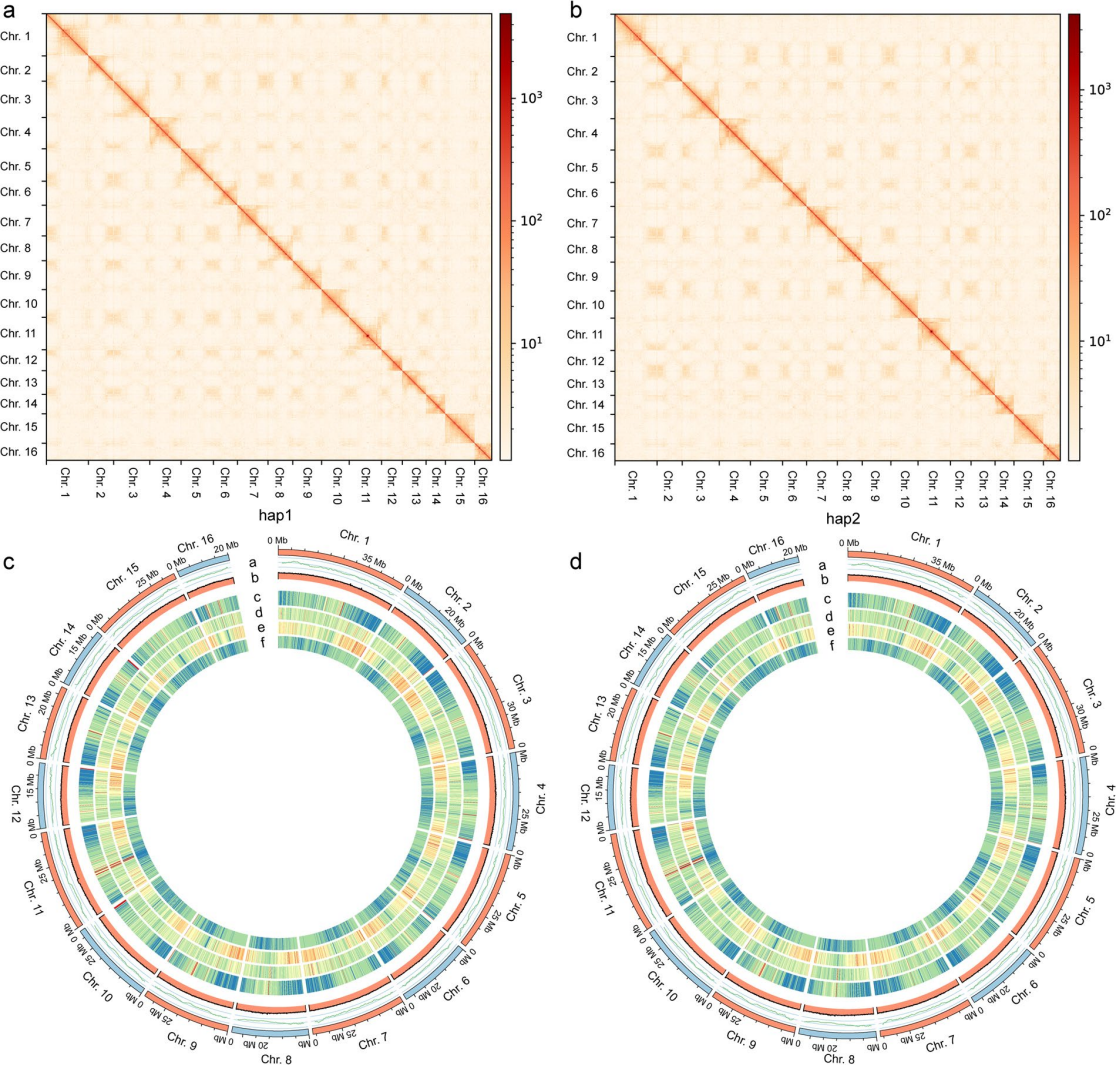
Hi-C heatmap and Circos plot for hap1 and hap2. (**a,b**) show the interaction links of the Hi-C data at a bin size of 20 kb. The interaction intensity is indicated using colours ranging from light yellow to dark red, which denote the frequency of Hi-C interactions from low to high. (**c,d**) Circos plot of the genomes of hap1 and hap2 with a window size of 500 kb. This Circos plot includes six components: (**a**) LAI (LTR assembly index) values; (**b**) density of GC content; (**c**) gene density; (**d**) density of *Copia* retrotransposons; (**e**) density of *Gypsy* retrotransposons; and (**f**) density of TEs (transposable elements).

After adaptor filtering, the HiFi reads were employed to assemble a primary contig dataset using hifiasm v0.16.1 with default parameters^52^. Second, all CCS reads were remapped to the above assembled contigs using minimap2 v2.24^53^, and the parameters were set as -secondary = n -cx map-pb. Third, Longshot (default settings) was used to call variations^54^. Fourth, variation phasing was realized by using WhatsHap with default parameters^55^. Fifth, CCS reads were separated into two haplotypes (hap1 and hap2) and an unassigned group according to the phased variations. Sixth, CCS reads of the hap1 combined unassigned group and hap2 combined unassigned group were assembled independently using hifiasm v0.16.1 with default parameters^52^. For genome scaffolding, clean Hi-C data were mapped to the contigs of hap1 and hap2 by BWA v0.7.10^56^. HiCExplorer^57^ was used to calculate mapping reads and interaction pairs. For hap1, a total of 120.89 million reads were uniquely mapped, of which 51.16 million were valid interactions, accounting for 42.32%. For hap2, 119.03 million reads were uniquely mapped, and 50.58 million reads were observed to interact with other pairs, for a percentage of valid data of 42.49%. Second, valid interaction read pairs were used to cluster and orient the assembled haplotype-resolved contigs onto pseudochromosomes by using HiCAssembler^58^ (options: -min_scaffold_length 100000; -bin_size 20000; -misassembly_zscore_threshold 1.0; -num_iterations 4; -num_processors 30). The Juicebox tool (https://github.com/aidenlab/Juicebox) was used to fine-tune the pseudochromosomes manually. This final assembly resulted in a total of 562.99 Mb (contig N50 = 34.10 Mb) assigned to 16 pseudochromosomes for hap1, of which 527.20 Mb could be oriented, accounting for 93.64% of the haplotype genome size, while 526.40 Mb of hap2 was oriented, accounting for 93.82% of the 561.07 Mb (contig N50 = 33.91 Mb) of the assembled sequences (Table 2; Fig. 2c,d). Heatmaps were produced to display the interactions of genome read pairs at a resolution of 20 kb (Fig. 2a,b).

**Table 2:** Overview of the haplotype-resolved genome assembly.

Assembly index	hap1	hap2
Genome before scaffolding by HiC		
Counts of Contig sequences	789	829
Largest contig length (bp)	38,200,000	38,210,000
Contig N50 (bp)	16,857,125	10,663,198
Contig N90 (bp)	2,804,056	1,680,000
Genome after scaffolding by HiC		
Counts of scaffold sequences	708	703
Length of scaffold sequences (bp)	562,990,894	561,068,552
Largest scaffold length (bp)	49,766,161	49,731,481
Scaffold N50 (bp)	34,103,335	33,910,957
Scaffold N90 (bp)	22,892,000	22,406,140
GC content (%)	36.71%	36.71%
N Length (bp)	162,000	252,000
N content (%)	0.03%	0.04%
Gap numbers	81	126

### Genome-wide repetitive sequence annotation

A comprehensive transposable element (TE) prediction tool, Extensive Denovo TE Annotator^59^ (options: -t 20 -step all -sensitive 1 -anno 1), was adopted to construct a nonredundant TE library with Helitron and long terminal repeat (LTR) identification. TIR-Learner v2.5^60^ was used to search for terminal inverted repeats (TIRs) under default recommendations. RepeatMasker v4.05^61^ was used to predict the final repeat sequences of the genome under the parameters -nolow -no_is -norna -engine wublast. Finally, we identified 46.26% and 46.23% repetitive elements in the hap1 and hap2 assemblies, respectively, including similar components of the LTR type (4%–8%), TIR type (0–5%), non-LRT type (~0.50%) and non-TIR type (2%–9%) between hap1 and hap2 (Table 3; Fig. 2c,d).

**Table 3:** Annotations of repeated sequences in hap1 and hap2.

Element	Class	hap1			hap2		
		Numbers	Length occupied (bp)	Percentage of sequence (%)	Numbers	Length occupied (bp)	Percentage of sequence (%)
LTR	Copia	49,843	36,934,468	6.56	52,452	38,093,854	6.79
	Gypsy	59,067	48,144,768	8.55	59,139	48,457,231	8.64
	Unknown	56,810	29,001,468	5.15	55,392	27,968,130	4.99
TIR	CACTA	69,243	26,040,568	4.63	72,898	27,068,332	4.83
	Mutator	72,764	20,818,845	3.7	72,396	20,322,553	3.62
	PIF_Harbinger	44,274	11,675,844	2.07	48,596	13,940,292	2.49
	Tc1_Mariner	4,654	1,481,269	0.26	4,706	1,325,219	0.24
	hAT	41,895	17,862,985	3.17	37,947	16,767,929	2.99
non LTR	LINE_element	5,825	2,521,684	0.45	5,421	2,396,560	0.43
nonTIR	Helitron	61,104	15,839,016	2.81	55,772	14,959,035	2.67
	Repeat_region	122,585	50,172,923	8.91	118,992	47,873,476	8.54
	Total	588,064	260,493,838	46.26	583,711	259,172,611	46.23

### Gene model prediction and functional annotation

Three steps were combined for protein-coding gene model prediction. First, AUGUSTUS v3.4.0^62^ and GeneMark-ES v4.68^63^ were used to search for gene models via *ab initio* prediction. Second, GeMoMa v1.3.1^64^ was used for homology prediction. Then, PASA (parameters: -align_tools gmap, -maxIntronLen 20000) was used for gene prediction via RNA-seq^65^. Finally, 41039 gene models were annotated for hap1, and 39744 gene models were annotated for hap2 (Table 4; Fig. 2c,d). For functional annotation, the above gene model sequences were translated into protein sequences and subsequently compared against the COG v2.1.4^66^, GO^67^, Pfam^68^ and KEGG^69^ databases. In total, 34617 hap1 and 34562 hap2 nonredundant gene models were annotated by these biological databases, accounting for 84.35% and 86.96% of all gene models, respectively (Table 4). The gene model numbers of hap1 and hap2 were greater than those reported in Chandler^26^ (32498), Serr (Payne × PI 159568)^70^ (31425) and Ding *et al*.^71^ (33430) and lower than those reported in Chandler^28^ (37554) and Zhongmucha-1^29^ (39432).

**Table 4:** Overview of the haplotype-resolved genome annotation.

Annotation index	hap1	hap2
Gene model annotation		
Number of gene models	41,039	39,744
Number of mRNA	43,309	42,373
Number of exon	213,870	215,844
Number of intron	170,536	173,431
Mean coding sequence length (bp)	1,093	1,107
Mean number of exons per mRNA	4.94	5.09
Mean exon length (bp)	221	217
Mean intron length (bp)	693	736
Non-coding RNA annotation		
tRNA	500	500
rRNA	72	104
miRNA	207	210
snoRNA	1,038	1,105
Functional annotation		
COG	32,341	32,267
GO	18,069	18,055
KEGG	17,123	17,291
Pfam	31,305	31,219
Total	34,617	34,562

### Noncoding RNA annotation

tRNAscan-SE v2.0.9^72^ (default settings), barrnap v0.9^73^ (options: -kingdom euk -threads 8) and Infernal v1.0^74^ (option: -Z $Z -cut_ga -rfam –nohmmonly -fmt 2) combined with Rfam^75^ were used for the tRNA, rRNA and miRNA/snoRNA searches, respectively. Overall, 1817 (500 tRNA, 72 rRNA, 207 miRNA and 1038 snoRNA) and 1919 (500 tRNA, 104 rRNA, 210 miRNA and 1105 snoRNA) noncoding RNAs were discovered in the genomes of hap1 and hap2, respectively (Table 4).

### Simple sequence repeat (SSR) recognition and variation calling

**Fig. 3:**
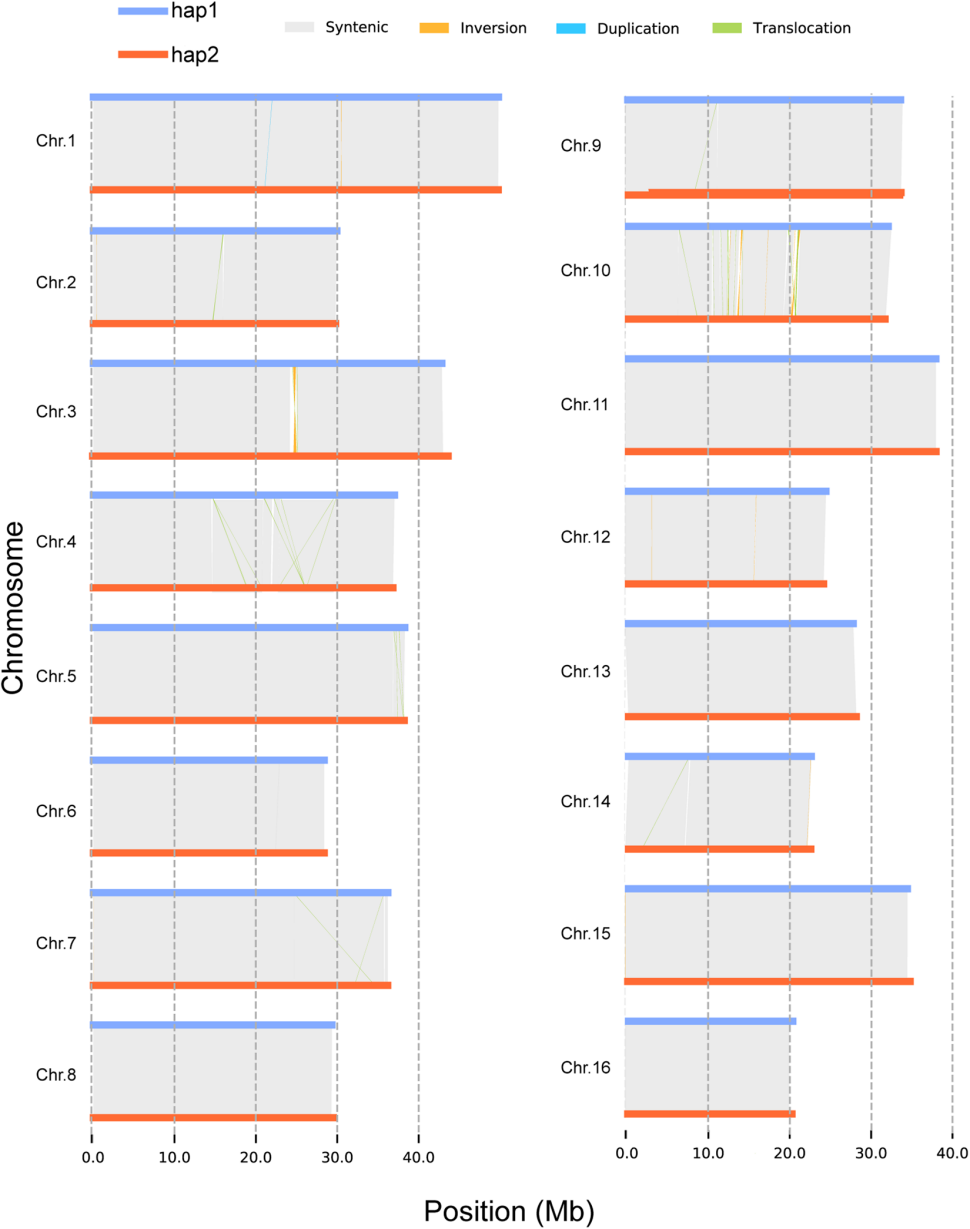
SV (structural variation) distribution between the haplotypes. The horizontal blue lines indicate the chromosomes of hap1, and the red lines indicate the chromosomes of hap1. The grey, orange, light blue and green lines indicate the syntenic, inverted, duplicated and trans-located regions, respectively.

Hap1 and hap2 were analysed by using TBtools v 1.098751^76^ for SSR recognition under default settings. We identified 358869 SSRs for hap1 and 358104 SSRs for hap2. In addition, we used hap2 as a query to map it to hap1 using minimap2 v2.24^53^ under the settings -eqx -ax asm5, followed by variation detection using SyRI^77^ under default settings. Afterwards, we detected 21 inversions, 40 translocations, 575 presence/absence variants (PAVs), 2 duplications in hap1 and 60 duplications in hap2 (Table 5; Fig. 3). Notably, the lengths of the PAVs were 274,629 bp in hap1 and 274.840 bp in hap2, accounting for 303 and 272 genes in hap1 and hap2, respectively (see repository files^78^). Similarly, the structural variations between hap1 and three released genomes^29,70,71^ of *Juglans* were identified (see repository files^78^).

**Table 5:** Structural variation and presence/absence variants between hap1 and hap2.

Variation type	Numbers	Length in hap1 (bp)	Length in hap2 (bp)
Inversions	21	1,037,925	1,090,802
Translocations	40	1,026,758	1,033,924
Duplications (hap1)	2	6,247	—
Duplications (hap2)	60	—	210,298
Presence/absence variant (>50 bp)	575	274,629	274,840

### Allele-specific expression genes (ASEGs) under cold stress

**Fig. 4:**
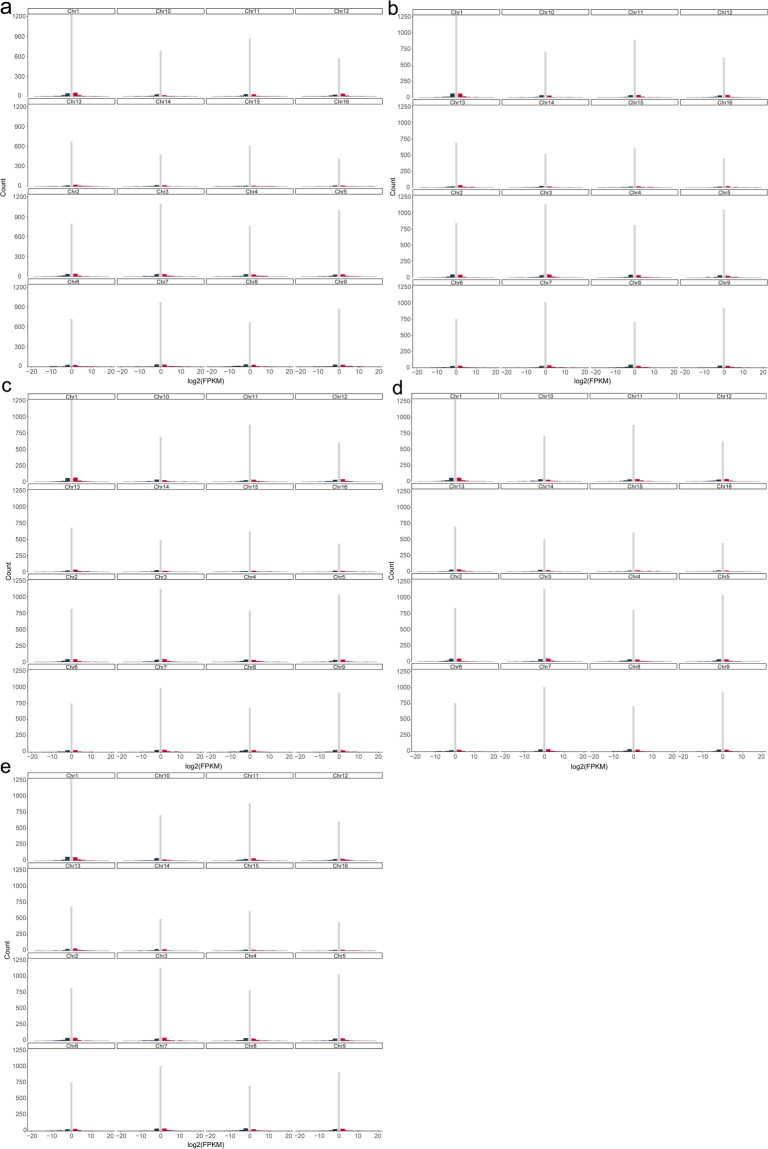
Histograms of the biallelic expression of divergent alleles in homologous chromosome pairs at 0 h (**a**), 3 h (**b**), 6 h (**c**), 12 h (**d**), and 24 h (**e**). The black column indicates hap1 dominance. The red column indicates hap2 dominance. The grey column indicates equal expression.

**Fig. 5:**
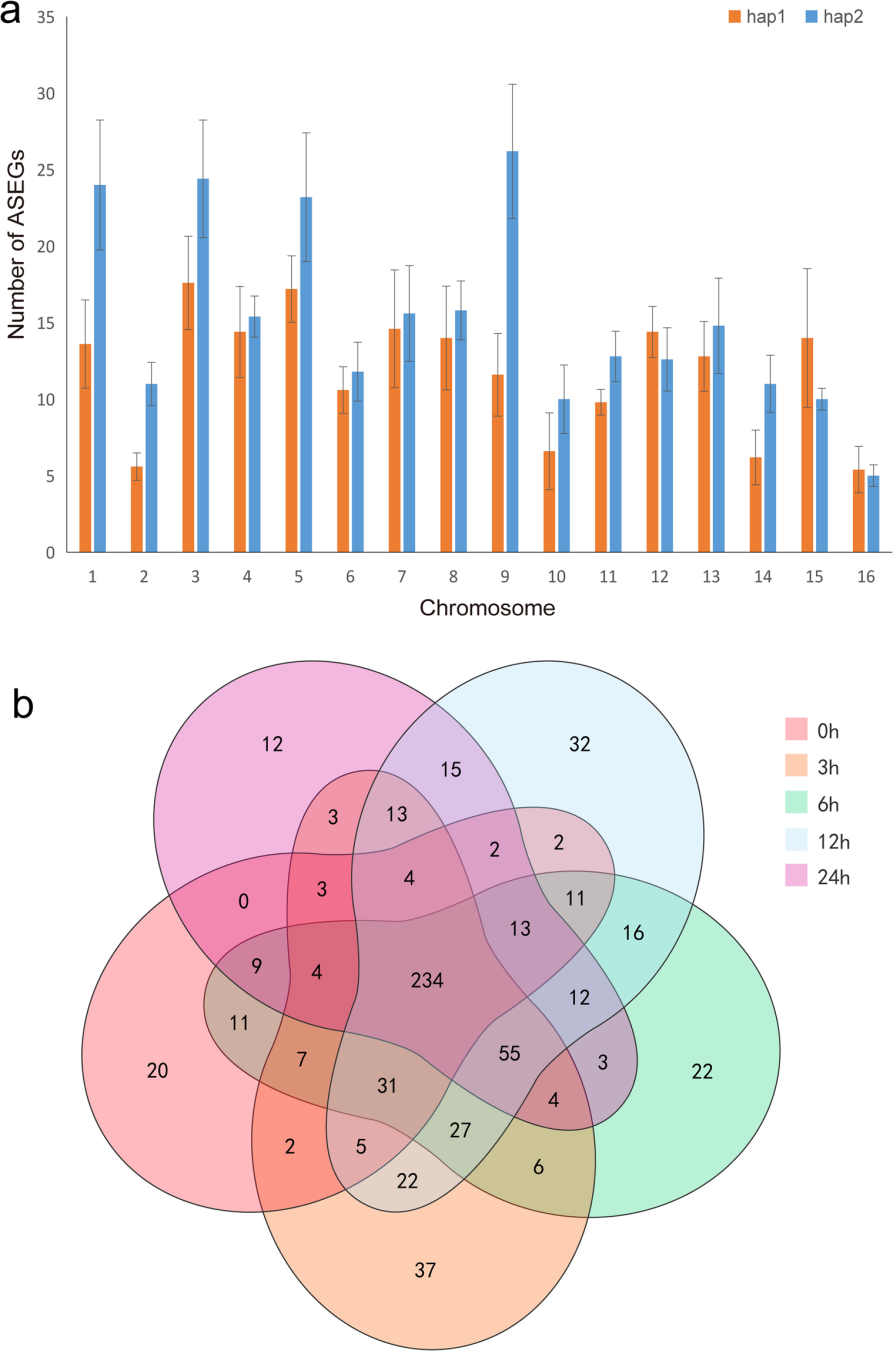
Allele-specific gene expression in hap1 and hap2 under cold stress. (**a**) Allele-specific expression genes (ASEGs) on haplotype chromosomes at five time points under cold stress (0 h, 3 h, 6 h, 12 h, 24 h). The numbers of ASEGs are displayed as the mean ± s.d. (**b**) Venn diagram of the ASEGs at five time points under cold stress. The numbers indicate unique and common ASEGs for different time points.

To explore the response of ASEGs to cold stress, three-leaf-stage seedlings derived from the kernels of wild walnut trees were treated at 4 °C in a climate chamber (Shanghai Boxun Industrial Co., Ltd., Shanghai, China). Leaves (approximately 0.5 g) were sampled after treatment for 0 h, 3 h, 6 h, 12 h and 24 h, with three biological repetitions, and immediately placed in liquid nitrogen for quick freezing. RNA-seq libraries were constructed, and sequencing was performed (see Methods: Plant material preparation, Illumina short-read library construction and sequencing). MCScanX^79^ was used to identify collinear block gene pairs between hap1 and hap2 with the default settings. The scaffold-involved collinear gene pairs were removed. Subsequently, the collinear gene pairs were checked by BLAST^80^ with a similarity between 90% and 100%. Ultimately, we obtained 18770 gene pairs. The clean RNA-seq data were mapped to the walnut genomes using HISAT2 v2.20^81^, and TPM values were used for expression quantification by StringTie v 2.1.2^82^. DESeq 2^83^ was used to determine ASEGs with the criteria |log_2_Fold Change| > 1 and adjusted *p < *0.05. We obtained 358, 457, 465, 494 and 386 ASEGs at 0 h, 3 h, 6 h, 12 h and 24 h, respectively, accounting for approximately 2% of all the identified collinear genes (see repository files^78^; Fig. 4). Of all the ASEGs, 234 were shared, and 12, 37, 22, 32 and 20 were specifically detected in the 0 h, 3 h, 6 h, 12 h and 24 h samples, respectively (Fig. 5b). We also noticed that more frequent ASEGs were dominantly expressed on hap2 (Fig. 5a), indicating uncoordinated allelic expression patterns for the ASEGs under cold stress in wild walnut.

## Data Records

Illumina reads for genome survey, RNA-seq reads and Hi-C reads were deposited in the Sequence Read Archive (SRA) (accession numbers: PRJNA858167, PRJNA859241, and PRJNA858917, respectively)^84–86^. HiFi long reads were co-deposited in the National Genomics Data Center (https://ngdc.cncb.ac.cn/) under the accession number CRA007543 and SRA under accession number PRJNA947329^87^. The genome assembly of wild walnut (*Juglans regia* L.) has been deposited on the Figshare platform (10.6084/m9.figshare.22266730)^88^ and GenBank with accession numbers GCA_034508915.1 and GCA_034509015.1, respectively^89,90^. The RNA-seq reads of cold-stressed samples were deposited in the SRA under accession number PRJNA942426^91^.

## Technical Validation

### Sequencing read quality control

For Illumina-based sequencing data (sequencing for genome survey, Hi-C reads and RNAseq reads), raw reads were removed to obtain clean data according to the following steps: (1) filtering adaptors; (2) removing reads with >10% unidentified nucleotides; and (3) removing reads with >50% bases with a Q value ≤ 10. The clean data were then evaluated in terms of the insert size, GC content, Q30 value, quality distribution and base composition. Specifically, we used HiCUP^92^ (v0.6.1) for preprocessing and to evaluate the Hi-C library quality. After trimming the restriction enzyme site of HindIII by calling the HiCUP_truncater script, the Hi-C clean data were aligned to the haplotype genomes by BWA v0.7.10^56^, and only the unique mapped sequences with a mapping score of at least 20 were used for the following process. For the HiFi long reads, subreads from the same template strand were consistently corrected, and adapters and low-quality reads were filtered. Before processing, 2000 randomly selected long reads were subjected to contamination evaluation by BLAST^80^ against the Nt database (ftp://ftp.ncbi.nih.gov/blast/db) under the threshold of *P* < 1e-05.

### Haplotype genome assembly evaluation

**Fig. 6:**
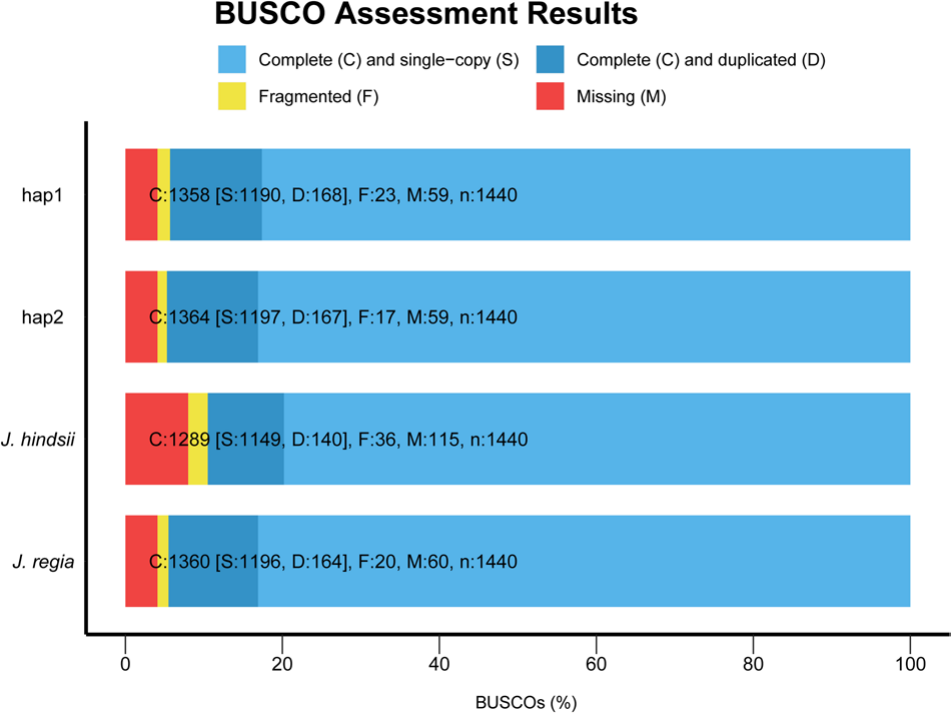
Summary assessment bar chart for BUSCO evaluation in hap1, hap2 and published genomes of *Juglans* (http://xhhuanglab.cn/data/juglans.html). Light blue indicates the percentage of complete and single-copy genes, while darker blue indicates the percentage of complete and duplicated genes; yellow indicates the percentage of fragmented genes; and red indicates the percentage of missing genes in the assemblies.

**Fig. 7:**
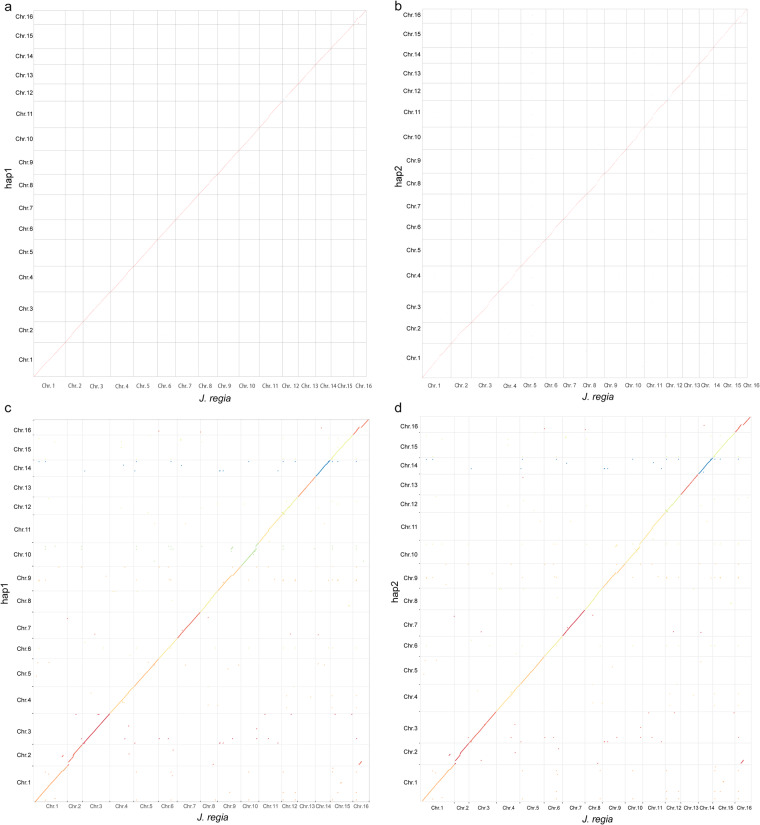
Collinearity evaluation between haplotypes and the published genome of *Juglans regia* L^29,71^. (**a**) The collinearity between hap1 and the published *Juglans regia* L. genome^29^. (**b**) The collinearity between hap2 and the published *Juglans regia* L. genome^29^. (**c**) The collinearity between hap1 and the published *Juglans regia* L. genome^71^. (**d**) The collinearity between hap2 and the published *Juglans regia* L. genome^71^. To distinguish the two published genomes, chromosome^71^ collinearity was marked using different colours.

BUSCO v5^45^ evaluation revealed that more than 94% of Embryophyta genes were successfully assembled in both hap1 and hap2 (Fig. 6), indicating that the completeness of these haplotype-resolved genomes was in line with that of the published genome^29^. The LAI^44^ values of hap1 and hap2 were 13.36 and 13.41, respectively. The CC ratios^93^ of hap1 and hap2 were 43.25 and 42.94, respectively. Furthermore, we used hap1 and hap2 as queries for mapping against two published genomes^29,71^ in minimap2 v2.24^53^ under the settings -eqx -ax asm5. Good collinearities were observed between our haplotype genomes and the two published genomes^29,71^ (Fig. 7). Finally, the phasing accuracy of the haplotype-resolved assembly was assessed by the percentage of switch errors using the modified calc_switchErr pipeline^32,94^. As 10x Genomics linked reads were not available, the criterion consistency phased SNP step was excluded from the phasing evaluation pipeline. A total of 184957 SNPs were detected, including 33061 switched SNPs, indicating a switch error rate of 17.87%, which is similar to that in the genome of *Solanum tuberosum* L. (17.1%) and lower than that in the genomes of *Malus domestica* cv. Gala (22.2%) and *Vanilla planifolia* (44.0%)^94^. We further used published Nanopore sequencing data (SRR10001245) from the *Juglans regia* L. cultivar Chandler^28^ to evaluate phasing accuracy. We first polished the ONT data using the genome resequencing data of wild walnut with Ratatosk^95^ v0.7.0 under default settings; then, the same method was used to calculate the switch error. Finally, we obtained a switch error of 6.25%. Collectively, these results indicate that a reference-level high-quality haplotype-resolved genome of wild walnut was obtained.

### Published QTL confirmation

To further validate the accuracy of the genome, we downloaded published reduced-representation sequencing data from *Juglans regia* L., which were used for high-density genetic map construction and QTL mapping^96^. A total of 2540 out of 2577 markers (98.56%), developed by specific length amplified fragment sequencing (SLAF-seq), on the genetic map can be mapped to the genome of hap1 with an identity >90% and an E threshold value < E-10 (see repository files^78^). Specifically, a QTL identified by the authors for anthracnose resistance harbouring 10 markers was found to be best mapped to chr1:14148922–14220999 of hap1, a region of approximately 721 kb (Table 6). A similar discovery was made for hap2. These results indicated the high accuracy of the subgenomes of this wild walnut.

**Table 6:** BLAST result of a published QTL containing 10 markers.

Query	Genetic distance (cM)	Chromosome ID of hap1	Identity (%)	Alignment length	Mismatches	Start	End	E value	Bit score
Marker101952	165.507	chr1	100	81	0	14159170	14159090	3.26E-35	150
Marker26540	166.266	chr1	97.647	85	0	14148922	14148838	1.51E-33	145
Marker8527	167.615	chr1	100	81	0	14172406	14172326	3.26E-35	150
Marker9161	168.937	chr1	100	80	0	14188508	14188587	1.17E-34	148
Marker39939	171.812	chr1	97.647	85	1	14221083	14220999	1.51E-33	145
Marker52721	172.643	chr1	100	80	0	14151361	14151282	1.17E-34	148
Marker13797	174.499	chr1	100	80	0	14214557	14214636	1.17E-34	148
Marker528341	175.105	chr1	100	41	0	14150797	14150837	2.30E-13	76.8
Marker51138	176.317	chr1	100	81	0	14165642	14165562	3.26E-35	150
Marker56359	176.332	chr1	98.81	84	0	14209546	14209463	1.17E-34	148

## Data Availability

No specific code was developed in this work. All the data were analyzed following the manuals suggested by the developers of the bioinformatic tools, which have been described in the Methods section.
